# Case report: Magnetocardiography as a potential method of therapy monitoring in amyloidosis

**DOI:** 10.3389/fcvm.2023.1224578

**Published:** 2023-08-17

**Authors:** Ainoosh Golpour, Phillip Suwalski, Ulf Landmesser, Bettina Heidecker

**Affiliations:** Deutsches Herzzentrum der Charité, Universitätsmedizin Berlin, Corporate Member of Freie Universität Berlin and Humboldt – Universität zu Berlin, Berlin, Germany

**Keywords:** magnetocardiography, amyloidosis, therapy response, treatment, screening, diagnosis, tafamidis, monitoring

## Abstract

Amyloidosis is characterized by a disorder of protein conformation and metabolism, resulting in deposits of insoluble fibrils in various organs causing functional disturbances. Amyloidosis can also affect the heart. Cardiac amyloidosis tends to have a poor prognostic outcome if diagnosed at a late stage. Therefore, early diagnosis and initiation of therapy as well as monitoring of treatment response are crucial to improve outcomes and to learn more about its pathophysiology and clinical course. We present an 83-year-old woman with cardiac transthyretin amyloidosis (ATTR) who was treated with tafamidis. The patient significantly improved 18 months after initiation of therapy with regards to exercise capacity and quality of life. In addition to standard diagnostic methods, we used magnetocardiography (MCG) to monitor potential treatment response by detecting changes in the magnetic field of the heart. MCG is a non-invasive method that detects the cardiac magnetic field generated by electrical currents in the heart with high sensitivity. We have recently shown that this magnetic field changes in various types of cardiomyopathies may be used as a non-invasive screening tool. We determined previously that an MCG vector ≥0.052 was the optimal threshold to detect cardiac amyloidosis. The patient's MCG was measured at various time points during therapy. At the time of diagnosis, the patient's MCG vector was 0.052. After starting therapy, the MCG vector increased to 0.090, but improved to 0.037 after 4 months of therapy. The MCG vector reached a value of 0.017 after 5 months of therapy with tafamidis, and then increased slightly after 27 months to a value of 0.027 (<0.052). Data from this case support our previous findings that MCG may be used to monitor treatment response non-invasively. Further research is needed to understand the unexpected changes in the MCG vector that were observed at the beginning of therapy and later in the course. Larger studies will be necessary to determine how these changes in the electromagnetic field of the heart are related to structural changes and how they affect clinical outcomes.

## 1. Introduction

Protein conformation and metabolism are dysfunctional in patients with amyloidosis, leading to the accumulation of protein deposits in various organs including the heart, liver, kidneys and gastrointestinal tract (1).

Due to advances in cardiovascular imaging, increasing awareness and age of the population, the prevalence of patients diagnosed with amyloidosis is rising (2). There are two main types of cardiac amyloidosis: Transthyretin amyloidosis (ATTR) and immunoglobulin light chain amyloidosis (AL). AL amyloidosis is characterized by the accumulation of amyloid protein in the heart and the kidneys and is often associated with clonal plasma cell disorders such as multiple myeloma (2, 3). ATTR amyloidosis is further divided into two subtypes: Wild type (ATTRwt) and variant (ATTRv). ATTRwt amyloidosis often affects the heart, causing symptoms such as shortness of breath and arrhythmias. This type of amyloidosis is often preceded by carpal tunnel syndrome or spinal canal stenosis, which are caused by amyloid deposits. ATTRv amyloidosis primarily affects the peripheral and autonomic nervous system (4). Patients with AL amyloidosis have a poorer prognosis than patients with ATTR amyloidosis, although the prognosis in patients with AL amyloidosis has improved significantly over time (2, 5).

It is important to accurately diagnose and classify the type of amyloidosis of a patient, as this will guide treatment (6). Tafamidis is the only treatment approved by the US Food and Drug Administration (FDA) for ATTR cardiomyopathy (7–9). Other ATTR stabilizers, such as diflunisal and acoramidis, are being investigated as potential treatments (8, 9). Furthermore, novel therapies are evaluated in clinical trials including gene silencers such as patisiran and inotersen, *in vivo* gene therapy with CRISPR cas, ATTR degraders, and anti-TTR antibodies (8–12). In some cases, liver transplantation is considered in ATTRv cardiomyopathy, as TTR is produced by the liver leading to a stop of main production of TTR after transplantation (13).

Heart involvement in amyloidosis can lead to heart failure and a range of clinical symptoms (2). Cardiac amyloidosis can cause restrictive cardiomyopathy, which leads to stiffening of the ventricles (14), and atrial fibrillation increasing the risk of atrial thrombus formation and thromboembolism (15–17). The diagnosis of cardiac amyloidosis can be made using non-invasive methods such as electrocardiography (ECG), echocardiography, cardiac magnetic resonance imaging (MRI), or Technetium-99m-labelled 3,3-diphosphono-1,2-propanodicarboxylic acid (99mTc-DPD) scintigraphy. Endomyocardial biopsy is the gold standard for definitive diagnosis of amyloidosis (2). Biomarkers such as N-terminal pro-brain natriuretic peptide (NT-proBNP) and cardiac troponin T (cTnT) are often elevated in patients with amyloidosis (13). Early diagnosis of amyloidosis is important to reduce the accumulation of amyloid deposits in organs and the risk for disease progression. While there have been significant advances in the treatment of cardiac amyloidosis in recent years, there continues to be a need for more effective therapeutic strategies and tools to monitor disease progression and therapy response. To address this issue, we used magnetocardiography (MCG) in a patient with ATTR amyloidosis who was treated with tafamidis.

## 2. Methods

MCG is a non-invasive contactless technique that detects the cardiac magnetic field produced by electrical currents in the heart (18). It is a safe and effective diagnostic tool, as it does not involve radiation exposure and can measure the magnetic field of the heart with high accuracy (10^−15^ to 10^−11^ Tesla) (18). The important components of the MCG instrument are the 64 highly sensitive magnetic sensors, known as Superconducting Quantum Interference Device Sensors (SQUIDs). These sensors are capable of detecting and measuring the magnetic field changes induced by the heart during the cardiac cycle. The device is located in a magnetically shielded room to reduce interference from external factors with electromagnetic properties. Electromagnetic interference signals can be compensated using various frequency filters. The MCG signals are a result of ionic flows that generate the potential differences measured by ECG. The measurement of the magnetic field involves a three-dimensional resolution where a sum vector can be created as the main electrical axis of the heart. The term MCG vector in our manuscript refers to the area of the green line formed by multiple vectors. For our measurements, we focused on the loop from T-begin to T-max (corresponding to the beginning and the maximum of the T-wave of an ECG). Specifically, we analyzed the MCG vector during the T-begin to T-max interval in the baseline and follow up examinations using the software BMP Cardio Expert Ver. 2.5.1. The analyzed time period was 60 s for each measurement. For more information on the exact determination of the MCG vector, please see the supplementary material provided in our last publication (18). During an MCG procedure, the patient is monitored through a 12-channel ECG. The entire measurement takes approximately 60 s, and data is collected using a multi-channel system that captures the entire thoracic magnetic field (18, 19). The magnitude and orientation of the heart's magnetic field are reflected by a sum vector that points towards the left shoulder in healthy hearts (20) and is located in the first quadrant on a MCG display (18). Pathologies of the heart can be detected by a score, and a change in the shape or position of the sum vector, e.g., if there is a shift from the 1st to the 2nd or 3rd quadrant (18). MCG may be a useful tool in the diagnosis and management of various cardiac conditions. In this case report MCG was used as a screening tool for amyloidosis and to monitor the patient's response to tafamidis therapy.

## 3. Case report

We report on an 83-year-old woman with ATTR amyloidosis. Relevant pre-existing conditions included heart failure with preserved ejection fraction (NYHA II) and status post MitraClip intervention. Medications on admission included standard heart failure therapy according to the guidelines of the European Society of Cardiology (ESC) (21).

She was admitted to the hospital for treatment of paroxysmal atrial fibrillation with rapid ventricular response unresponsive to metoprolol.

The patient reported limited exercise tolerance during the last year. The patient described dyspnea on exertion when walking two flights of stairs. She denied syncope or presyncope. Blood pressure was normal on admission and, according to the patient, in general well controlled (average 130/70 mmHg over the last few months). Physical examination and laboratory results including complete blood count and complete metabolic panel were unremarkable. 12-lead ECG confirmed atrial fibrillation without conduction defect or ST segment abnormalities.

**Figure 1:**
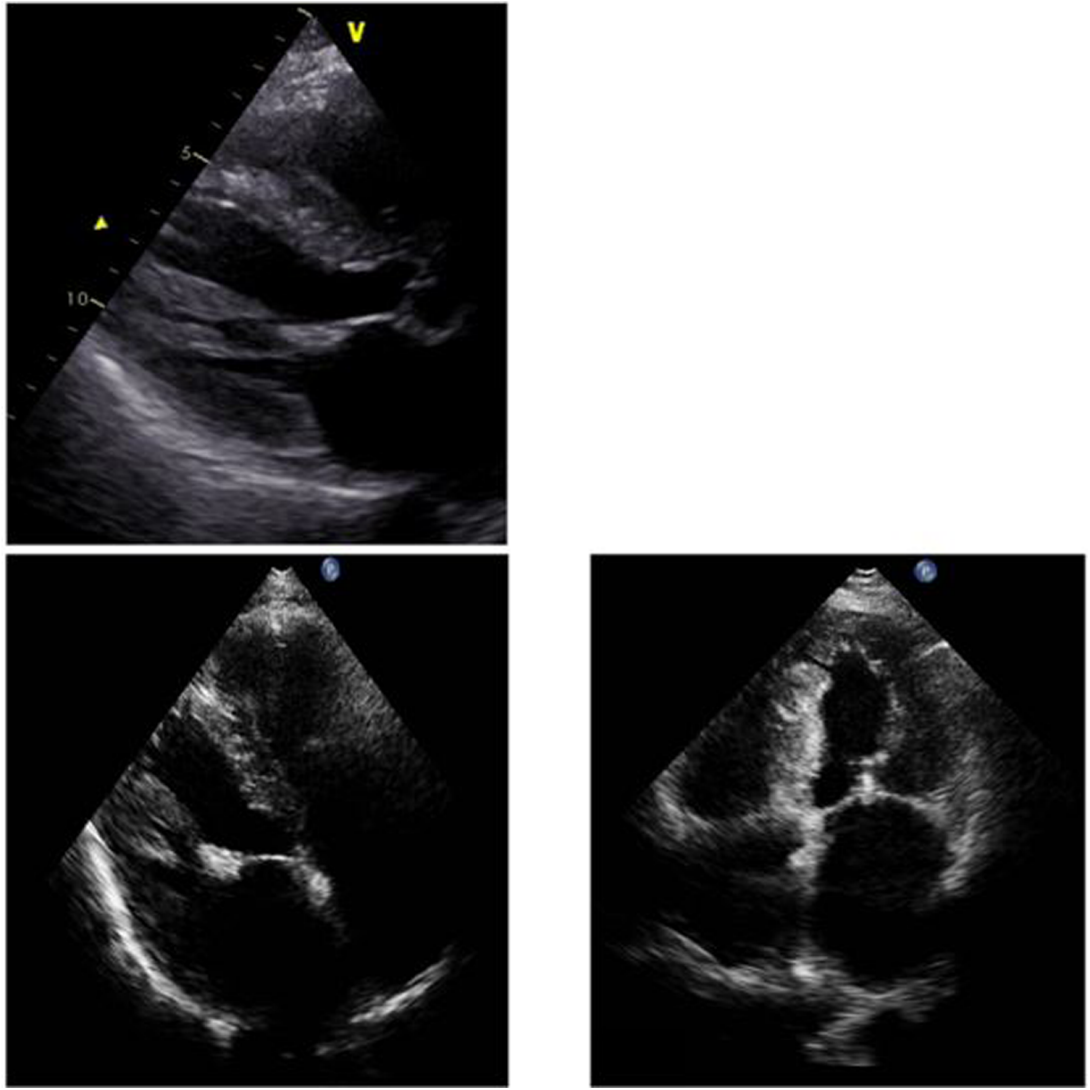
Myocardial thickening and dilated atria in the parasternal long axis and in the apical four chamber view.

During her hospital stay, the patient was evaluated with endomyocardial biopsy for suspected amyloidosis. Echocardiogram demonstrated left ventricular thickening, preserved left ventricular systolic ejection fraction (LVEF 60%), a dilated left atrium (left atrial volume 94 ml), minimal residual mitral valve regurgitation (status post MitraClip placement) and normal left ventricular size (Figure 1). There were no wall motion abnormalities. The systolic pulmonary artery pressure was 41 mmHg.

**Figure 2:**
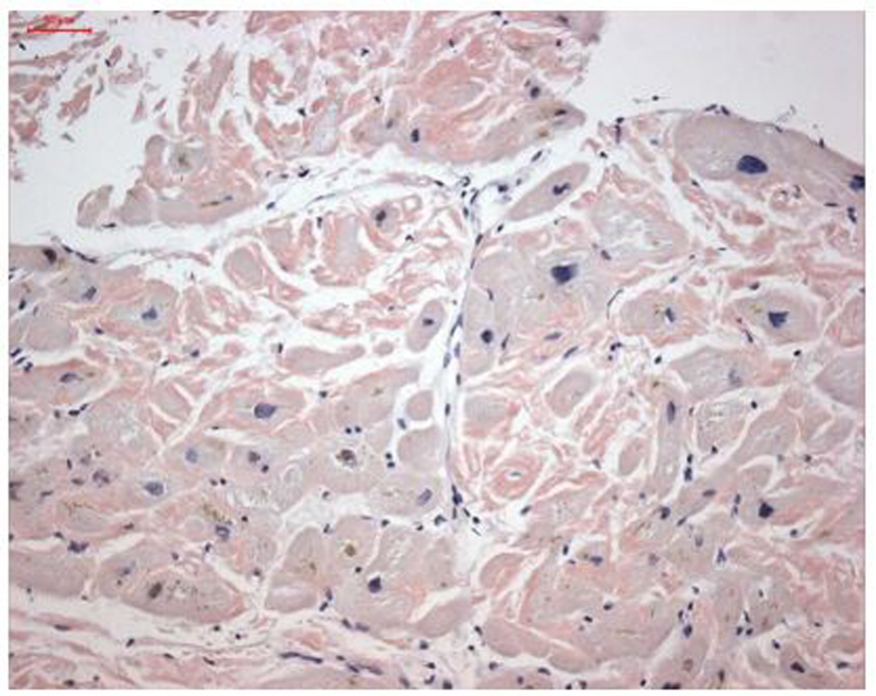
Amyloid deposits in the patient's biopsy. Courtesy of Dirk Lassner, IKDT.

Endomyocardial biopsy revealed cardiac TTR deposits (Figure 2). Genetic analysis was negative for ATTRv. Therefore, the final diagnosis was ATTRwt. Monoclonal light chains were excluded in serum and urine, supporting the diagnosis of ATTRwt and the absence of multiple myeloma or AL amyloidosis.

**Figure 3:**
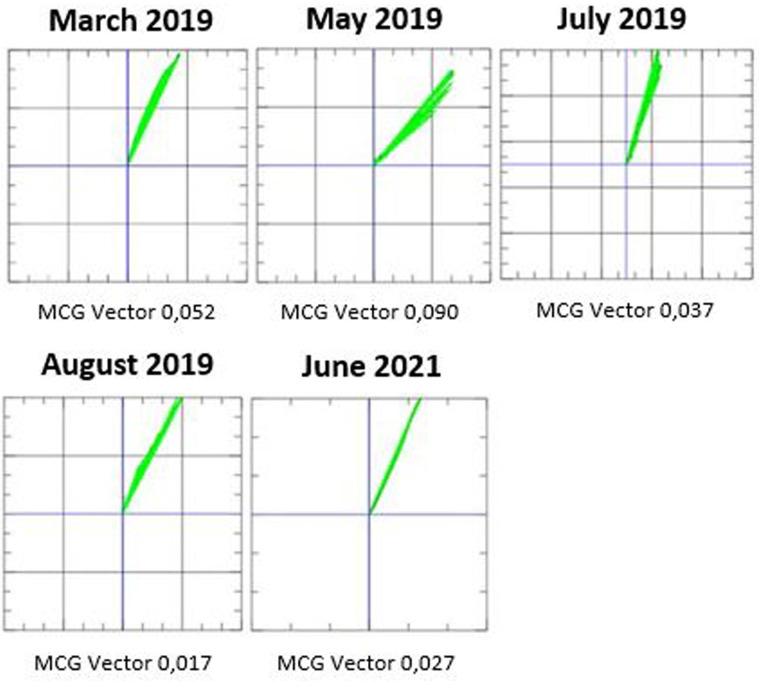
Magnetocardiography (MCG) measurements from March 2019 to June 2021 during treatment with tafamidis for ATTRwt amyloidosis.

The patient was followed in our outpatient clinic, where therapy with tafamidis was initiated in addition to standard heart failure therapy. Before initiation of therapy, an MCG measurement was performed and was found to be pathological with a value of 0.052 (Figure 3). After that, we started therapy with tafamidis 20 mg per os daily and followed the patient every 2–3 months with physical examination, laboratory tests, 12-lead ECG, 24 h-ECG, echocardiography, and MCG. A chronological overview of the MCG measurements over the course of 2 years is provided in Figure 3.

We recently demonstrated an MCG vector of 0.052 as the optimal cut point value for the diagnosis of amyloidosis (18). Also in our patient the initial MCG vector was 0.052 before initiation of therapy. In the second measurement, the MCG vector increased towards more pathological values (0.090). However, after 4 months therapy, the vector normalized to 0.037. The MCG vector remained within normal range for several months and reached a value of 0.017 after 5 months of therapy. After 27 months of treatment, the MCG vector increased slightly, but was still normal at 0.027. We increased the dose of tafamidis to 61 mg after 18 months (new dosage recommendation was issued) (22); the patient reported an improvement in exercise tolerance after increasing the dose of tafamidis. Furthermore, there was an overall improvement in the patient's quality of life during therapy with tafamidis 61 mg daily.

ECGs from all of the patient's follow-up examinations showed deep S-spikes in V3–V6 with a delayed R/S transition that was more pronounced at the follow-up examination from May 2019. However, there were no new T-wave, T-amplitude, or ST-segment changes, and there was no correlation between the patient's ECG and MCG findings. The sensitivity of ECG and MCG is different, for example, ECG is sensitive to currents that are radial to the chest surface, whereas MCG is sensitive to tangential currents (23).

**Figure 4:**
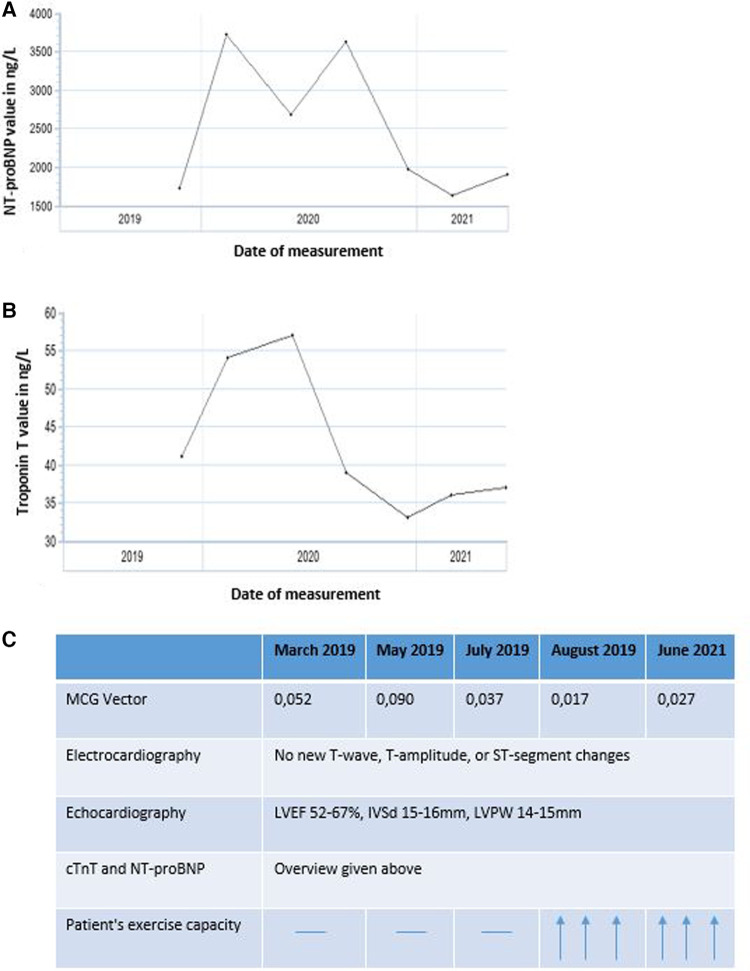
Overview of (**A**) NT-proBNP (ng/L) and (**B**) cardiac troponin T (cTnT, ng/L) levels from February 2019 to July 2021 as well as (**C**) summary of the time-variation for all parameters.

During therapy, LVEF ranged from 52% to 67%, with no discernible trend. Interventricular septal thickness (IVSd) and left ventricular posterior wall thickness (LVPW) were between 15 and 16 mm and 14–15 mm, respectively, before and during therapy. Performance of longitudinal strain analysis was unfortunately limited in this patient due to atrial fibrillation. cTnT and NT-proBNP serum levels were abnormal with cTnT ranging from 33 to 57 ng/L and NT-proBNP ranging from 1,730 to 3,621 ng/L between visits (Figure 4), with an overall downward trend and intermittent peaks. In late 2019 to mid-2021, the patient reported feeling increasingly better, which also correlated with the decrease of cTnT and NT-proBNP levels, as well as with the decrease of the MCG vector. The renal parameters creatinine and urea were minimally elevated before initiation of therapy with a value of 1.1 and 50 mg/dl, respectively, and remained stable during the course of treatment. There were no relevant changes in other laboratory parameters.

## 4. Discussion

Since publication of the ATTRACT study in 2018, cardiac amyloidosis has been increasingly diagnosed, given that there is now a novel, effective therapy for patients with ATTR cardiomyopathy (7). According to a recent study, between 2000 and 2012, the prevalence rate for cardiac amyloidosis overall increased from 8 to 17 per 100,000 person-years and the incidence rate from 18 to 55 per 100,000 person-years (24). Cardiac amyloidosis is a slowly progressive disease. Early, targeted therapy is important to reduce the risk of progression and increases the chance for therapy success (7). This case report demonstrates an improvement of the MCG vector calculated from the interval T-begin to T-max during therapy with tafamidis measured by MCG. MCG could become a valuable tool to monitor treatment response noninvasively in non-ischemic cardiomyopathies (18). Larger studies are needed to test that hypothesis.

To date, the role of MCG has been investigated primarily in early diagnosis of ischemic heart disease (25–29). MCG has been reported as a potential diagnostic tool in coronary artery disease and myocardial infarction (30). A two-center study investigated the value of MCG in the evaluation of ischemia (31). In addition, MCG has been tested for the diagnosis of cardiac arrhythmias (32–34). It has also been applied during various provocation tests (35), including drug infusion, exercise stress tests (36, 37), or cardiac pacing (38), in an attempt to enhance diagnostic accuracy for ischemic cardiac diseases and arrhythmias. Brockmeier and coworkers reported that also early signs of diabetic cardiomyopathy can be identified in the MCG (39), while Korhonen et al. detected repolarization abnormalities in patients with dilated cardiomyopathy and ventricular arrhythmias using MCG (40). Furthermore, studies have shown a potential role of MCG in the diagnosis of left ventricular overload and hypertrophy (41–43).

In a recent study involving 14 groups of patients with different cardiac conditions (including healthy patients, athletes, patients with microvascular disease, ischemic heart disease, and patients with left ventricular hypertrophy), a new method for determining the patient's cardiac condition using MCG was proposed with a reported sensitivity of 70% and a specificity of 98% (44). This new method included current density distribution maps of the various groups (44). By analyzing current vector maps, it is possible to gain information about distribution of electrical currents in the heart and abnormalities related to different cardiac conditions. Studies of current density vector map classification based on cluster analysis in patients with coronary artery disease and ischemic heart disease who had normal or nonspecifically altered ECG and echocardiogram have previously yielded promising results (45, 46).

Early reports investigated inflammation of the myocardium associated with myocarditis and heart transplant rejection with MCG (47–49). In our recent work, we have shown that diseases such as inflammatory cardiomyopathy and amyloidosis can be detected with MCG (17). Those findings were more pronounced in acute inflammatory cardiomyopathy, but an effect could also be seen in amyloidosis. Furthermore, we demonstrated in a small cohort that MCG was able to detect treatment response within 7 days of therapy in inflammatory cardiomyopathy. The specificity of MCG to detect non-ischemic cardiomyopathy overall was 95%, while the sensitivity was 59% similar to what had been reported in the past (18, 48). Furthermore, we demonstrated that MCG was able to detect mRNA vaccine-associated myocarditis, which is a rare but potentially serious adverse event associated with mRNA vaccination (50). Furthermore it has been shown that MCG could potentially be used to visualize myocardial damage in COVID-19 patients (23).

The role of ECG, body surface potential mapping, and vectorcardiography for comparative evaluation versus magnetocardiography in detecting nonischaemic cardiomyopathies require further investigation. A study has shown that 87-lead body surface potential mapping in patients with idiopathic dilated cardiomyopathy can identify individuals at risk for sustained ventricular tachycardia with a sensitivity of 73% and a specificity of 76% (51). Vectorcardiography has already been proposed as a tool to detect scar in patients with nonischaemic cardiomyopathy and to identify patients at risk of arrhythmias (52). In nonischaemic cardiomyopathy, the ECG is used in diagnostic staging and risk stratification. The specificity of ECG is somewhat limited (53). For example, the presence of a fragmented QRS complex has been reported to be predictor of arrhythmic events in patients with nonischaemic cardiomyopathies (54). However, fragmentation of QRS complexes is encountered also in coronary artery diseases and is not specific for subtypes cardiomyopathy (54). Moreover, a study proposed that signal-averaged ECG allows identification of patients with nonischaemic dilated cardiomyopathy and sustained ventricular tachycardia with a sensitivity of 80% and a specificity of 66% (55). In this case report, we suggest that response to therapy in a patient with amyloidosis may be monitored by MCG. The MCG vector of this patient may have been affected by tafamidis therapy, by standard heart failure therapy, or both. We observed an increase in the MCG vector immediately after the start of therapy and at the last measurement. Many scenarios could explain these fluctuations. Various changes of the heart structure may lead to possible changes of the MCG vector. We have recently shown that the positive predictive value of MCG is high (93%) (18). However, the method is limited in its inability to distinguish between different pathologies. Amyloidosis is a disease in which therapy is a long-term process and fluctuations, including changes of cardiac enzymes, are commonly observed during therapy. The patient's cardiac wall thickness remained unchanged despite therapy, while there was improvement in the MCG vector. Tafamidis is primarily designed to stabilize transthyretin protein and prevent further amyloid deposition. Currently, cardiac improvement and therapy response in amyloidosis are detected by echocardiography, including longitudinal strain measurements at follow up examinations amongst other functional testing (56). Our data suggest that MCG is a valuable tool to monitor therapy response. Further research is needed to determine its role as a screening tool and to study its role to detect therapy response in a larger cohort.

MCG has multiple advantages to be used in diagnostic screening and therapy response: it can be performed quickly and the results can be evaluated immediately. There are no known side effects and the examination is non-invasive. If MCG were to be introduced as a screening tool in hospitals detection of a pathological vector would facilitate diagnostic workup to detect cardiomyopathies such as inflammatory cardiomyopathy or amyloidosis early and initiate treatment in a timely manner.

## 5. Conclusion

The following conclusions emerge from this case report:

• MCG is a valuable screening method to detect pathologies of the heart and may support the diagnosis of amyloidosis and other cardiomyopathies with the help of established diagnostic methods. In this case report, the MCG vector improved during treatment with tafamidis and standard heart failure therapy. MCG could be a potential monitoring tool for therapy response in cardiac amyloidosis.
• The causes of the initial increase in the MCG vector after initiation of therapy with tafamidis and the small increase at the last measurement are currently unclear. It may be helpful to collect more data on a larger number of patients to identify any patterns or trends that could shed light on the underlying causes of these changes.
• Further studies are needed to determine the exact role of MCG in therapy monitoring, as well as in the diagnosis of cardiac amyloidosis.

## Data Availability

The raw data supporting the conclusions of this article will be made available by the authors, without undue reservation.
